# A predictive model for early death in elderly colorectal cancer patients: a population-based study

**DOI:** 10.3389/fonc.2023.1278137

**Published:** 2023-12-18

**Authors:** Qi Wang, Kexin Shen, Bingyuan Fei, Hai Luo, Ruiqi Li, Zeming Wang, Mengqiang Wei, Zhongshi Xie

**Affiliations:** Department of Gastrointestinal Colorectal and Anal Surgery, China-Japan Union Hospital of Jilin University, Changchun, China

**Keywords:** early death, nomogram, colorectal cancer, SEER (surveillance epidemiology and end results) database, elderly patients

## Abstract

**Purpose:**

The purpose of this study is to determine what variables contribute to the early death of elderly colorectal cancer patients (ECRC) and to generate predictive nomograms for this population.

**Methods:**

This retrospective cohort analysis included elderly individuals (≥75 years old) diagnosed with colorectal cancer (CRC) from 2010-2015 in the Surveillance, Epidemiology, and End Result databases (SEER) databases. The external validation was conducted using a sample of the Chinese population obtained from the China-Japan Union Hospital of Jilin University. Logistic regression analyses were used to ascertain variables associated with early death and to develop nomograms. The nomograms were internally and externally validated with the help of the receiver operating characteristic curve (ROC), calibration curve, and decision curve analysis (DCA).

**Results:**

The SEER cohort consisted of 28,111 individuals, while the Chinese cohort contained 315 cases. Logistic regression analyses shown that race, marital status, tumor size, Grade, T stage, N stage, M stage, brain metastasis, liver metastasis, bone metastasis, surgery, chemotherapy, and radiotherapy were independent prognostic factors for all-cause and cancer-specific early death in ECRC patients; The variable of sex was only related to an increased risk of all-cause early death, whereas the factor of insurance status was solely associated with an increased risk of cancer-specific early death. Subsequently, two nomograms were devised to estimate the likelihood of all-cause and cancer-specific early death among individuals with ECRC. The nomograms exhibited robust predictive accuracy for predicting early death of ECRC patients, as evidenced by both internal and external validation.

**Conclusion:**

We developed two easy-to-use nomograms to predicting the likelihood of early death in ECRC patients, which would contribute significantly to the improvement of clinical decision-making and the formulation of personalized treatment approaches for this particular population.

## Introduction

Currently, colorectal cancer (CRC) ranks as the third most prevalent form of malignancy and stands as the second primary contributor to cancer-related mortality (1). As the population ages, there is sure to be a matching increase in the number of elderly colorectal cancer (ECRC)patients. Based on previous research,42% of CRC cases were those who aged older than 75 years, and for patients aging older than 55 years, the incidence of CRC would increase by approximately 30% for every 5-year increase in age (2, 3). Survival rates of CRC patients have significantly improved due to improvements in diagnosis and treatment, while overall survival rates of ECRC patients continue to be low (4). ECRC Patients frequently encounter mortality within a span of three months after their initial diagnosis (early death). There could be a few causes for this phenomenon: colonoscopy is the most effective colorectal cancer screening method, reducing incidence and mortality of disease (5). The effect of colonoscopy depends on the quality of bowel preparation (6). An excessive number of elderly individuals endure colonoscopies with inadequate bowel preparation, which reduce on the accuracy of Colonoscopy outcomes and potentially result in misdiagnosis or failure to diagnose CRC (7, 8). Moreover, compared to young people, elderly people have poor physical conditions and multiple comorbidities, making it difficult for them to tolerate systematic treatment including surgery and chemotherapy (9, 10). In short, ECRC patients are at an increased risk of experiencing early death as a result of delayed initial detection and restricted therapeutic efficacy (11, 12).

Nowadays, the tumor-node-metastasis (TNM) stage system of American Joint Committee on Cancer (AJCC) is the established standard for determining the best course of treatment and prognosis for CRC patients. Yet, in actual reality, CRC patients with comparable TNM stages exhibit considerable prognostic variations as well as diverse responses to treatments, underscoring the shortcomings of the TNM staging system for precise therapy of CRC (13). This might be due to the fact that in addition to biological elements, non-biological components also exert an influence on the prognosis of CRC (14). The elderly population is more susceptible to experiencing widowhood, which is considered a non-biological factor that has negative consequences for the prognosis of ECRC (15). Moreover, the incidence of Postoperative complications higher among ECRC patients, exerting a more pronounced impact on this population compared to younger patients with CRC (16). Therefore, the utilization of standard survival analysis in the treatment of older patients may introduce substantial bias. It is necessary to develop a new model to predict the incidence of early death in such patients. Nomograms which enable the estimation of the likelihood of a specific clinical event by integrating multiple prognostic variables are commonly employed as prognostic models in clinical practice (17). Clinicians can use nomogram to swiftly determine which ECRC patients have a greater chance of early death and create tailored clinical therapies for them.

In the present study, we intended to perform a retrospective population-based study to identify high-risk factors that are linked to early death and construct two nomograms that may be utilized to assess the likelihood of all-cause and cancer-specific early death in patients with ECRC.

## Methods

### Recruitment of patients from the SEER database

**Figure 1:**
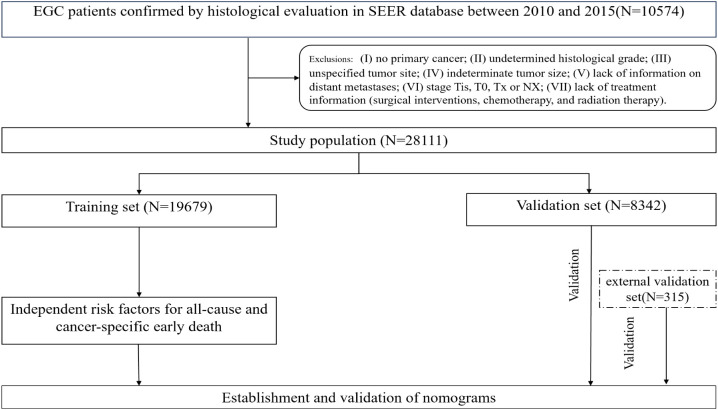
The patient selection procedure and the process involved in this research.

The Surveillance, Epidemiology, and End Results(SEER) database was a publicly accessible cancer database that encompasses information pertaining to the diagnosis, treatment, and survival outcomes of more than 8 million cancer cases throughout 18 states within the United States (18). The SEER database has been extensively utilized to examine the incidence and prognosis of numerous cancers, including early death of lung cancer, breast cancer, and other tumors, due to its substantial sample size and comprehensive follow-up information (19, 20). The present investigation constitutes a retrospective population-based study that relied on the use of the SEER database. In the SEER database, the collection of data on metastatic locations in bone, liver, lung, and brain did not commence until 2010. Consequently, this study included patients (≥75 years old) with a confirmed pathological diagnosis of CRC who were enrolled between 2010 and 2015 and successfully completed the follow-up. The identification of ECRC patients was conducted using the criteria outlined in the 3rd edition of the International Classification of Diseases for Oncology (ICD‐O‐3) based on the tumor primary site (C18.0-C18.7, C19.9, C20.09). Individuals who matched the subsequent criteria were not included: (I) no primary cancer; (II) undetermined histological grade; (III) unspecified tumor site; (IV) indeterminate tumor size; (V) lack of information on distant metastases; (VI) stage Tis, T0, Tx or NX; (VII) lack of treatment information (surgical interventions, chemotherapy, and radiation therapy). Finally, a total of 28,111 ECRC patients were enrolled and then separated into two cohorts using a random allocation method (7:3). The training cohort consisted of 19,679 patients, while the internal validation group included 8,432 patients. The research used publicly accessible data from the SEER database, and all individuals included in the study were anonymized. Consequently, the study was exempt from the need for ethics committee approval and informed consent. For the aim of external validation, data on 315 patients was collected retrospectively from the China-Japan Union Hospital at Jilin University between July 2017 and September 2022. A follow-up period of three months will be allocated to each patient. In the event that the patient succumbs to mortality within a span of three months after the initial diagnosis, we will duly document this occurrence. The last follow-up was in January of 2023. Similar selection procedures were used for the Chinese cohort as were used for the SEER cohort. The retrospective investigation of the Chinese cohort was approved by the ethics committee of the China-Japan Union Hospital at Jilin University in accordance with the principles articulated in the Helsinki Declaration. We use the same definition of early death as the published literature, which is death within three months following a diagnosis of CRC (20, 21). The endpoints were early death from all causes and cancer-specific early death. All-cause early death means that someone dies from any cause within three months of being diagnosed with CRC. Cancer-specific early death refers to death caused directly by the tumor itself within three months of being diagnosed with CRC, including death induced by any complications attributed to the treatment of the primary tumor. **Figure 1** shows the patient selection procedure and the process involved in this research.

### Clinical variables extracted for analysis

The extraction of factor pertaining to early death in ECRC patients is accomplished through consult available clinical documents (22, 23). The analysis extracted various factors related to patients, tumors, and treatment. These factors included patients’ characteristics such as gender, race, and year of diagnosis. Additionally, tumor and treatment characteristics such as tumor type, pathologic grade, T stage, N stage, M stage, primary tumor site, distant metastatic site, chemotherapy, surgery and radiotherapy were considered. Furthermore, survival data including follow-up time, survival status, and cause of death were also included in the analysis. The race was categorized as black or white and other. Histological type of ECRC patients is classified into four subtypes based on the ICD-O-3oncology code: adenocarcinoma (8140), mucinous adenocarcinoma (8480), signet ring cell adenocarcinoma (8490), and other types. Other types refer to pathological types other than those mentioned above, including soft issue tutors and sarcomas, acinar cell neoplasms, etc (24). The primary tumor site was divided into three groups: left colon, right colon, and rectum. A higher histologic grade represented a higher malignancy and was classified as follows: I-II and III-IV. Tumor size is defined as the largest cross-sectional diameter of the tumor, in previous studies, patients were divided into tumor ≤5 and tumor >5 cm groups, with 5 cm serving as the cut-off value (25, 26).

### Statistical analyses

The sample population concluded from SEER database was randomly split into a training set and a validation set with a proportion of 7 to 3 ratios using R software. Chi-square (χ2) was used to access the baseline variables of SEER cohort and Chinese cohort used for external validation cohort. Univariate logistic regression was used to determine how significantly different factors contributed to the early death of ECRC patients in the study’s validation cohort. The statistically significant variables were further investigated using multivariate logistic regression analysis. Using the risk factors that were shown to be statistically significant in the multivariate analysis, a prognostic nomogram was developed to estimate the likelihood of early death. Odds ratios (ORs) and 95% confidence intervals (CIs) were computed to determine the connection between clinical traits and the incidence of early death. The discriminating ability of the nomograms was measured by their area under the ROC curve. The precision of the nomograms was test using calibration curves. DCA was used to probe the nomogram’s clinical usefulness. ROC, calibration curves, and DCA were used to carry out internal validations.

Before conducting external validate, we determined the true cause of death of Chinese patients who were the external validation cohort through telephone follow-up. Given too many patients’ families were unable to give accurate information about the actual cause of death, external validation was exclusively performed on the all-cause early death nomogram in order to uphold the research’s rigor and objectivity. The results of external validation were also represented by ROC curves, calibration curves, and DCA curves.

Data were extracted using SEER*Stat 8.3.5 software (http://seer.cancer.gov/seerstat/). The statistical analysis was performed using R 3.5.2 (http://www.r-project.org) and SPSS 21.0 (IBM Corporation, Armonk, NY, USA) software. Mean ± standard deviation (SD) was used to describe the quantitative data; number and percentage (N, %) were used to describe these categorical data. A significant difference was deemed to exist when the p-value was less than 0.05 (95% CI). (Nakagawa and Cuthill 2007).

## Result

### Characterization of included cases

According to the criteria set forth for inclusion and exclusion, the current research covered a cohort of 28,111 seniors who suffered from CRC between the years 2010 and 2015 from the SEER database. Among the 28,111 senior individuals included in the study, a total of 3,500 (12.45%) patients experienced early death, of which 2494 (69.68%) patients succumbed to causes directly related to cancer, whereas 1085(30.32%) individuals passed away due to causes unrelated to cancer. Following is a brief overview of the clinical characteristics of these patients: 41.66% were male and 58.34% were female; 55.41% were unmarried and 44.59% were married; 99.61% were insured and 0.39% were uninsured; 73.37% had adenocarcinoma, 8.11% had mucinous adenocarcinoma, 1.00% had signet ring cell carcinoma and 17.52% had other types of cancer; 96.03% underwent surgery and 3.97% did not; 89.01% had colon cancer and 10.99% had rectal cancer; 0.31% of cases had bone metastasis, 0.09% had brain metastases, 6.88% had liver metastases, and 1.90% had lung metastases.

A total of 315 patients from our medical center were used as an external validation cohort, of whom 49 individuals (15.56%) experienced early death. Except for race, the Chi-square test revealed no significant differences between the patients from SEER and Chinese cohorts in other variables we extracted for analysis. There is a significant difference in the variable of race between the two cohorts, which may be due to our external validation cohort exclusively included Chinese population. The majority of cases in the Chinese cohort (N=232,73.65%) have adenocarcinoma, which matches up to that of the SEER cohort. In terms of tumor grade, grade I-II differentiation was most prevalent (N=247,78.41%). The liver was found to be the most common site for metastasis (N=22,6.89%). Further details regarding the clinical characteristics of patients with ECRC are presented in **Table 1**.

**Table 1:** Demographic and clinicopathological characteristics of ECRC patients with or without early death.

Variables	SEER cohort (n, %)					Chinese Cohort (n, %)	p
	Total N=28111	Training cohort N=19679	Inclusion Validation N=8432			External Validation N=315	
Insurance status							
Insured	28000 (99.61)	19605 (99.62)	8395 (99.56)			314 (99.68)	0.726
Uninsured	111 (0.39)	74 (0.38)	37 (0.44)			1 (0.32)	
Marital status							
Married	12536 (44.59)	8794 (44.69)	3742 (44.38)			141 (44.76)	0.891
Unmarried	15575 (55.41)	10885 (55.31)	4690 (55.62)			174 (55.24)	
Gender							
Female	16401 (58.34)	11513 (58.50)	4888 (57.97)			184 (58.41)	0.707
Male	11710 (41.66)	8166 (41.50)	3544 (42.03)			131 (41.59)	
Race							
Black	2140 (7.61)	1488 (7.56)	652 (7.73)			0 (0)	0
Other ^b^	2376 (8.45)	1666 (8.47)	710 (8.42)			315 (100)	
White	23595 (83.94)	16525 (83.97)	7070 (83.85)			0 (0)	
Primary site							
Left	17326 (61.63)	12114 (61.56)	5212 (61.81)			194 (61.59)	0.966
Rectum	3090 (10.99)	2154 (10.95)	936 (11.10)			34 (10.79)	
Right	7695 (27.37)	5411 (27.50)	2284 (27.09)			87 (27.62)	
Grade							
Grade I-II	22155 (78.81)	15479 (78.66)	6676 (79.17)			247 (78.41)	0.614
Grade III-IV	5956 (21.19)	4200 (21.34)	1756 (20.83)			68 (21.59)	
Histology							
Adenocarcinoma	20624 (73.37)	14515 (73.76)	6109 (72.45)			232 (73.65)	0.364
Mucinous adenocarcinoma	2281 (8.11)	1583 (8.04)	698 (8.28)			25 (7.94)	
Other	4924 (17.52)	3381 (17.18)	1543 (18.30)			56 (17.78)	
Signet ring cell carcinoma	282 (1.00)	200 (1.02)	82 (0.97)			2 (0.63)	
T stage							
T1	2808 (9.99)	1942 (9.87)	866 (10.27)			31 (9.84)	0.076
T2	4406 (15.67)	3088 (15.69)	1318 (15.63)			49 (15.56)	
T3	16001 (56.92)	11224 (57.04)	4777 (56.65)			159 (50.48)	
T4	4896 (17.42)	3425 (17.40)	1471 (17.45)			76 (24.13)	
N stage							
N0	17322 (61.62)	12033 (61.15)	5289 (62.73)			194 (61.59)	0.092
N1	6930 (24.65)	4902 (24.91)	2028 (24.05)			71 (22.54)	
N2	3859 (13.73)	2744 (13.94)	1115 (13.22)			50 (15.87)	
M stage							
M0	25185 (89.59)	17594 (89.40)	7591 (90.03)			282 (89.52)	0.295
M1	2926 (10.41)	2085 (10.60)	841 (9.97)			33 (10.48)	
Surgery							
No	1116 (3.97)	763 (3.88)	353 (4.19)			12 (3.81)	0.472
Yes	26995 (96.03)	18916 (96.12)	8079 (95.81)			303 (96.19)	
Chemotherapy							
No/Unknown	22703 (80.76)	15912 (80.86)	6791 (80.54)			255 (80.95)	0.821
Yes	5408 (19.24)	3767 (19.14)	1641 (19.46)			60 (19.05)	
Radiotherapy							
No/Unknown	26340 (93.70)	18449 (93.75)	7891 (93.58)			295 (93.65)	0.871
Yes	1771 (6.30)	1230 (6.25)	541 (6.42)			20 (6.35)	
Tumor size							
≤5cm	17744 (63.12)	12461 (63.32)	5283 (62.65)			202 (64.13)	0.532
>5cm	10367 (36.88)	7218 (36.68)	3149 (37.35)			113 (35.87)	
Bone metastasis							
No	28023 (99.69)	19616 (99.68)	8407 (99.70)			314 (99.68)	0.948
Yes	88 (0.31)	63 (0.32)	25 (0.30)			1 (0.32)	
Brain metastasis							
No	28085 (99.91)	19661 (99.91)	8424 (99.91)			314 (99.68)	0.434
Yes	26 (0.09)	18 (0.09)	8 (0.09)			1 (0.32)	
Liver metastasis							
No	26178 (93.12)	18320 (93.09)	7858 (93.19)			293 (93.02)	0.954
Yes	1933 (6.88)	1359 (6.91)	574 (6.81)			22 (6.98)	
Lung metastasis							
No	27576 (98.10)	19297 (98.06)	8279 (98.19)			309 (98.10)	0.776
Yes	535 (1.90)	382 (1.94)	153 (1.81)			6 (1.90)	

a Includes single, separated, widowed, and divorced.

b Includes American Indian/Alaska Native and Asian or Pacific Islander.

### Risk factor analysis for early death

Factors connected with early death in ECRC patients were ascertained using univariate and multivariate logistic regression analysis. Univariate logistic regression analysis revealed that race, marital status, tumor size, T stage, N stage, M stage, surgery, chemotherapy, radiotherapy, lung metastasis, brain metastasis, bone metastasis, and liver metastasis were all associated with all-cause and cancer-specific early death. Sex was only a high-risk factor for all-cause early death, whereas the factor of insurance status was solely associated with an increased risk of cancer-specific early death.

All these significant risk factors (p<0.05) identified in the univariate logistic regression analysis were imported into the multivariate logistic regression analyses. The findings of the study indicated that race, marital status, tumor size, Grade, T stage, N stage, M stage, brain metastasis, liver metastasis, bone metastasis, surgery, chemotherapy, and radiotherapy were independent prognostic factors for all-cause and cancer-specific early death in ECRC patients; The variable of sex was only associated with an increased risk of all-cause early death, whereas insurance status was solely only attached to an increased risk of cancer-specific early death. **Tables 2**, **3** include specific information.

**Table 2:** The univariate and multivariate logistic regression analyses of all-cause early death.

Variables		Univariate analysis				Multivariate analysis			
		OR	95%CIs		P-value	OR		95%CIs	P-value
Insurance status									
Insured		Reference				Reference			
Uninsured		1.44	0.79-2.63		0.232	NA		NA	NA
Sex									
Female		Reference				Reference			
Male		1.11	1.02-1.2		0.018	1.42		1.29-1.57	<0.001
Race									
Black		Reference				Reference			
Other ^a^		0.66	0.54-0.82		<0.001	0.67		0.53-0.85	0.001
White		0.87	0.74-1.01		0.06	0.95		0.81-1.13	0.581
Histology									
Adenocarcinoma		Reference				Reference			
Mucinous adenocarcinoma		0.92	0.78-1.07		0.269	0.91		0.77-1.08	0.3
Signet ring cell carcinoma		1.38	0.96-2		0.085	0.77		0.52-1.17	0.221
Other		0.81	0.72-0.92		0.001	0.96		0.85-1.1	0.593
Marital status									
Married		Reference				Reference			
Unmarried ^b^		1.46	1.34-1.59		<0.001	1.45		1.31-1.6	<0.001
Grade									
I-II		Reference				Reference			
III-IV		1.86	1.7-2.04		<0.001	1.39		1.24-1.55	<0.001
T stage									
T1		Reference				Reference			
T2		0.72	0.59-0.87		0.001	1.11		0.89-1.38	0.363
T3		1.12	0.96-1.31		0.158	1.37		1.13-1.66	0.002
T4		2.56	2.17-3.02		<0.001	2.28		1.84-2.82	<0.001
N stage									
N0		Reference				Reference			
N1		1.45	1.31-1.6		<0.001	1.56		1.39-1.75	<0.001
N2		2.59	2.32-2.88		<0.001	2.32		2.03-2.65	<0.001
M stage									
M0		Reference				Reference			
M1		3.96	3.57-4.4		<0.001	2.55		2.07-3.14	<0.001
Tumor size									
<5 cm		Reference				Reference			
≥5 cm		1.48	1.36-1.61		<0.001	1.11		1.01-1.23	0.029
Bone metastasis									
No		Reference				Reference			
Yes		9.12	5.53-5.05		<0.001	2.84		1.5-5.41	0.001
Brain metastasis									
No		Reference				Reference			
Yes		23.76	7.82-2.25		<0.001	11.76		2.69-51.35	0.001
Liver metastasis									
No		Reference				Reference			
Yes		3.76	3.33-4.25		<0.001	1.39		1.1-1.74	0.005
Lung metastasis									
No		Reference				Reference			
Yes		3.57	2.88-4.44		<0.001	1.24		0.93-1.65	0.139
Chemotherapy									
No		Reference				Reference			
Yes		0.16	0.13-0.2		<0.001	0.08		0.06-0.09	<0.001
Radiotherapy									
No		Reference				Reference			
Yes		0.3	0.22-0.39		<0.001	0.4		0.28-0.57	<0.001
Surgery									
No		Reference							
Yes		0.32	0.27-0.37		<0.001	0.15		0.12-0.19	<0.001
Primary site									
Left		Reference				Reference			
Rectum		0.66	0.57-0.77		<0.001	0.99		0.81-1.2	0.917
Right		1	0.91-1.1		0.954	1.05		0.95-1.17	0.318

a Includes American Indian/Alaska Native and Asian or Pacific Islander.

b Includes single, separated, widowed, and divorced.

**Table 3:** The univariate and multivariate logistic regression analyses of cancer-specific early death.

Variables	Univariate analysis			Multivariate analysis		
	OR	95%CIs	P-value	OR	95%CIs	P-value
Insurance status						
Insured	Reference			Reference		
Uninsured	1.98	1.07-3.69	0.031	2.07	1.05-4.08	0.035
Sex						
Female	Reference			Reference		
Male	1.01	0.92-1.12	0.826	NA	NA	NA
Race						
Black	Reference			Reference		
Other ^a^	0.68	0.53-0.87	0.002	0.69	0.52-0.91	0.009
White	0.87	0.73-1.03	0.115	0.98	0.81-1.2	0.871
Histology						
Adenocarcinoma	Reference			Reference		
Mucinous adenocarcinoma	0.88	0.73-1.06	0.191	0.87	0.71-1.06	0.168
Signet ring cell carcinoma	1.45	0.96-2.2	0.08	0.71	0.45-1.14	0.155
Other	0.75	0.65-0.86	<0.001	0.93	0.79-1.09	0.386
Marital status						
Married	Reference			Reference		
Unmarried ^b^	1.52	1.37-1.68	<0.001	1.34	1.2-1.49	<0.001
Grade						
I-II	Reference			Reference		
III-IV	2.15	1.94-2.39	<0.001	1.44	1.27-1.64	<0.001
Primary site						
Left	Reference			Reference		
Rectum	0.71	0.59-0.85	<0.001	1.06	0.83-1.34	0.652
Right	1	0.89-1.11	0.968	1.11	0.98-1.25	0.109
T stage						
T1	Reference			Reference		
T2	0.66	0.51-0.85	0.002	1.21	0.9-1.63	0.205
T3	1.32	1.08-1.61	0.007	1.72	1.33-2.21	<0.001
T4	3.59	2.92-4.41	<0.001	3.07	2.34-4.01	<0.001
N stage						
N0	Reference			Reference		
N1	1.73	1.54-1.95	<0.001	1.64	1.44-1.88	<0.001
N2	3.52	3.12-3.97	<0.001	2.66	2.28-3.1	<0.001
M stage						
M0	Reference			Reference		
M1	5.51	4.92-6.16	<0.001	3.04	2.45-3.78	<0.001
Tumor size						
≤5 cm	Reference			Reference		
>5 cm	1.69	1.53-1.86	<0.001	1.18	1.05-1.32	0.004
Bone metastasis						
No	Reference			Reference		
Yes	10.71	6.52-17.6	<0.001	2.48	1.33-4.65	0.005
Brain metastasis						
No	Reference			Reference		
Yes	26.73	9.52-75.06	<0.001	9.81	2.66-36.23	0.001
Liver metastasis						
No	Reference			Reference		
Yes	5	4.39-5.69	<0.001	1.42	1.12-1.79	0.004
Lung metastasis						
No	Reference			Reference		
Yes	4.57	3.65-5.73	<0.001	1.24	0.92-1.66	0.155
Chemotherapy						
No	Reference			Reference		
Yes	0.21	0.17-0.26	<0.001	0.09	0.07-0.11	<0.001
Radiation						
No	Reference			Reference		
Yes	0.38	0.28-0.51	<0.001	0.45	0.3-0.67	<0.001
Surgery						
No	Reference			Reference		
Yes	0.27	0.23-0.32	<0.001	0.13	0.1-0.17	<0.001

a Includes American Indian/Alaska Native and Asian or Pacific Islander.

b Includes single, separated, widowed, and divorced.

### Establishment and validation of the nomogram

**Figure 2:**
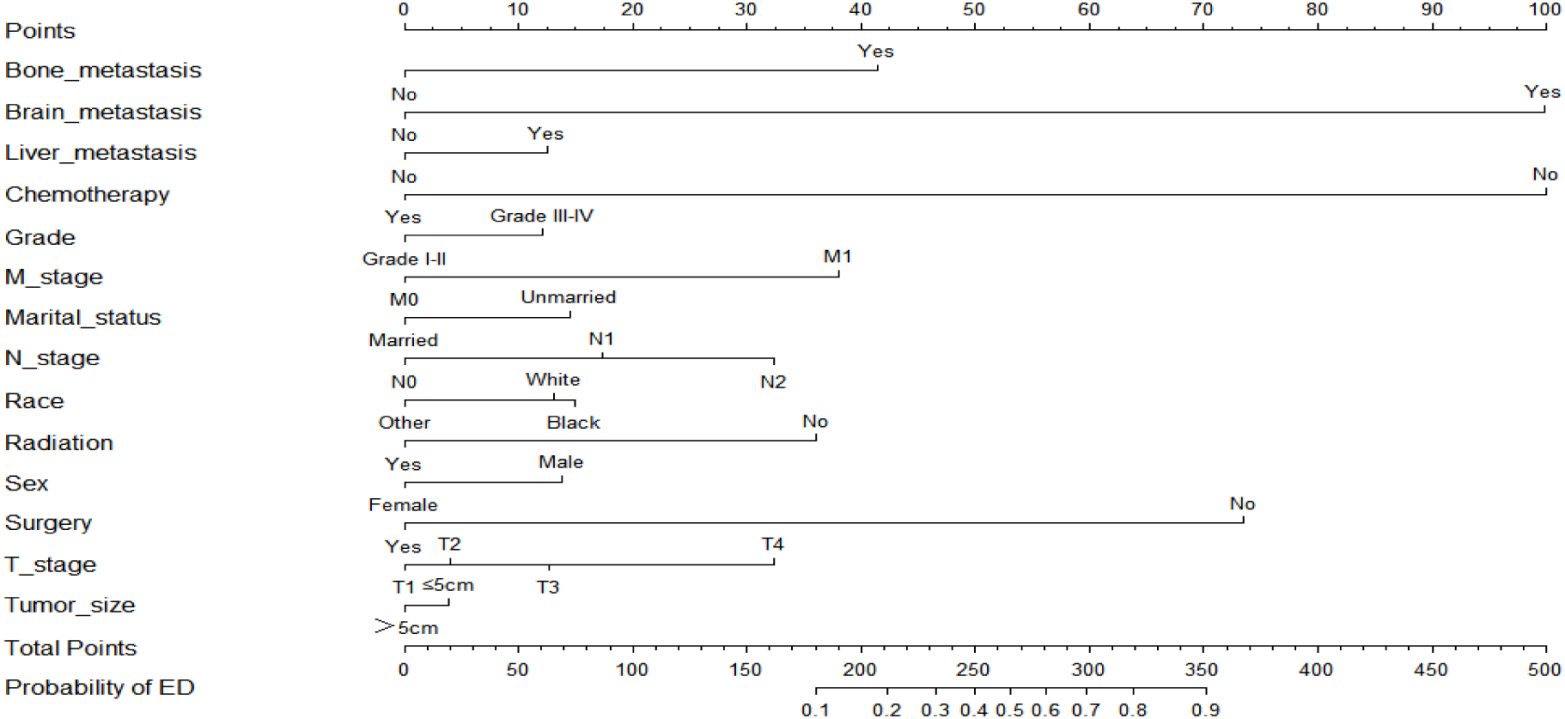
Nomogram for predicting all-causes early death.

**Figure 3:**
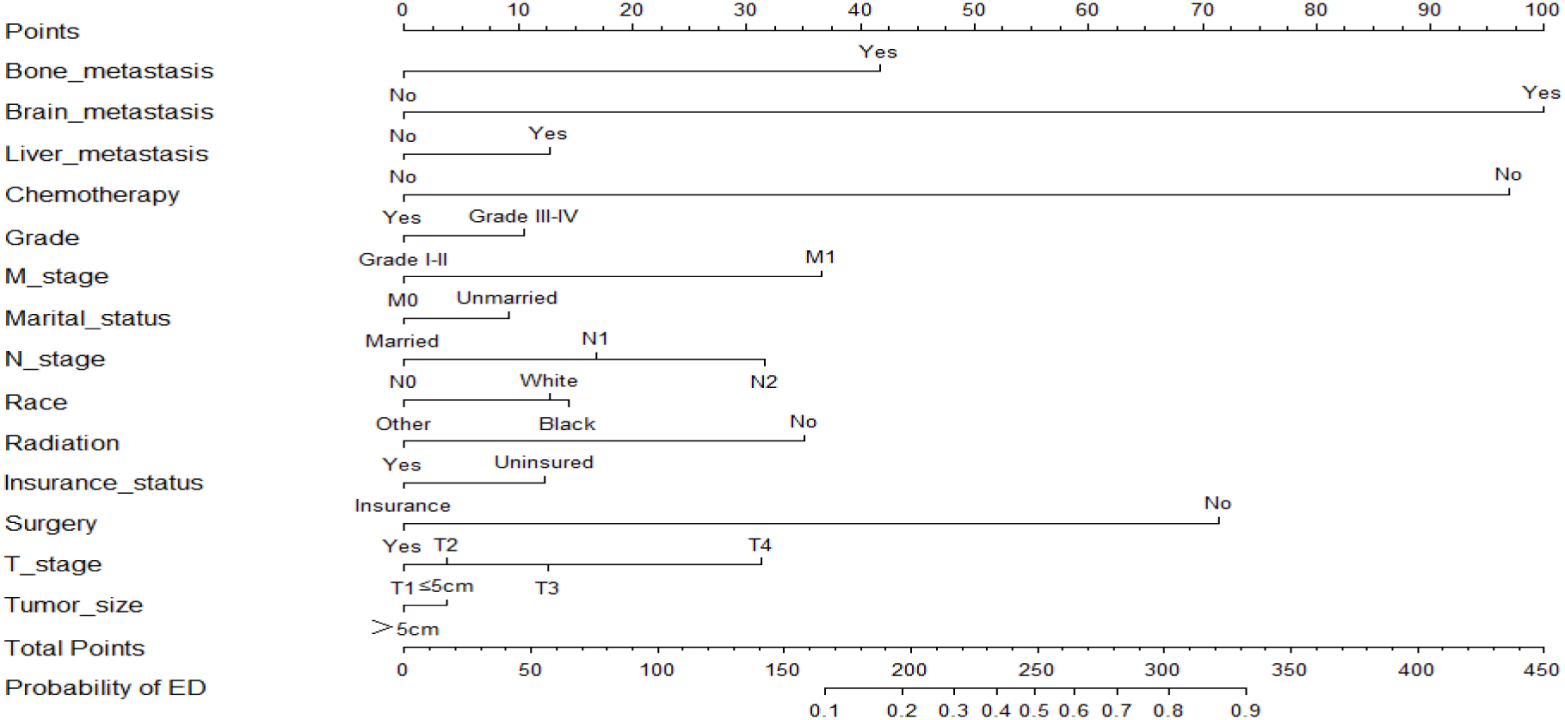
Nomogram for predicting cancer-specific early death.

Based on the results of the multivariate logistic regression analysis, two nomograms were developed to predict the risk of all-cause early death (**Figure 2**) and cancer-specific early death (**Figure 3**) in ECRC patients, respectively.

**Figure 4:**
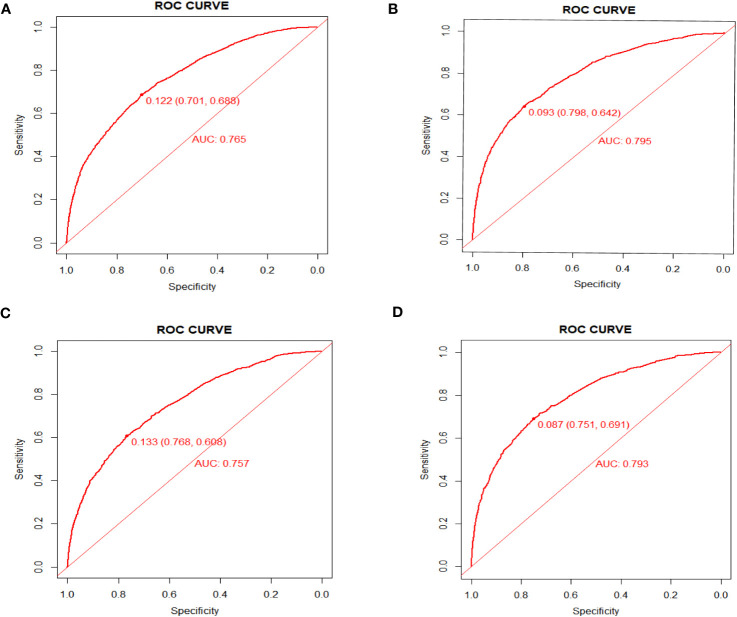
ROC curves for nomograms in predicting all-cause early death and cancer-specific early death in the training cohort **(A, B)** and the validation cohort **(C, D)**.

**Figure 5:**
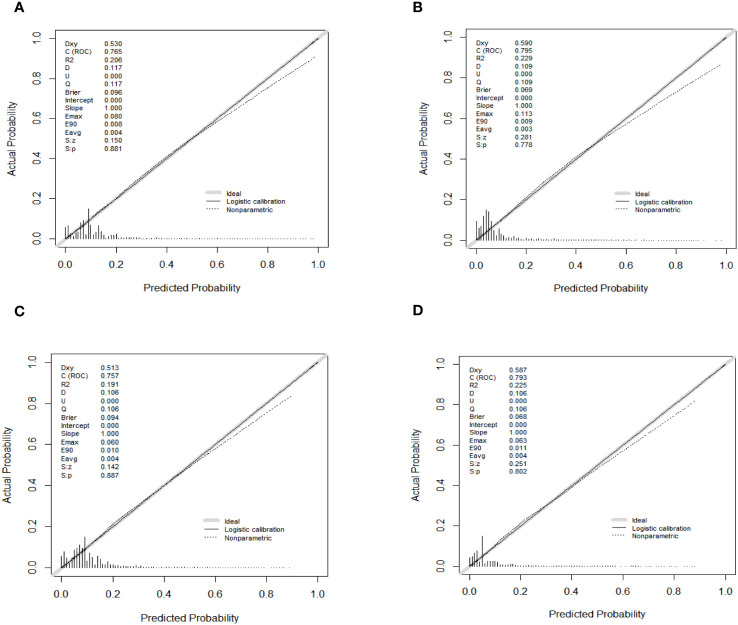
Calibration curves for nomograms in predicting all-cause early death and cancer-specific early death in the training cohort **(A, B)** and the validation cohort **(C, D)**.

**Figure 6:**
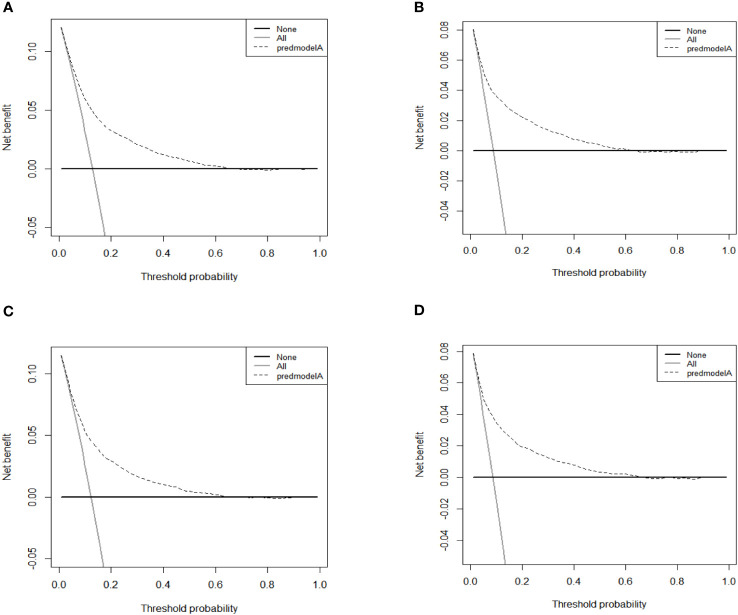
DCA curves for the nomograms in predicting all-causes early death and cancer-specific early death in the training cohort **(A, B)** and the validation cohort **(C, D)**.

As shown in **Figure 4**, the AUC values of the nomograms predicting the probability of all-cause early death and cancer-specific early death in the training cohort were 0.765 (95% CI: 0.7553-0.7751) and 0.795 (95% CI: 0.7836 - 0.8062), respectively. In the internal validation cohort, the AUC values of the nomograms for predicting early death from all causes and cancer-specific early death were 0.757 (95% CI: 0.741-0.7725) and 0.793 (95% CI: 0.776-0.8106), respectively. All nomograms demonstrated a satisfactory discriminatory ability. The horizontal axis of calibration curves reflects the estimated early death probability, whereas the ordinate represents the true probability. The anticipated and actual curves fit together, indicating that the nomograms exhibit a high level of consistency (**Figure 5**). In addition, the results of the DCA curves indicate that the innovative model exhibits a favorable positive net benefit and has excellent clinical utility (**Figure 6**). All of these findings of internal validation demonstrate the validity and scientific rigor of our prediction model.

**Figure 7:**
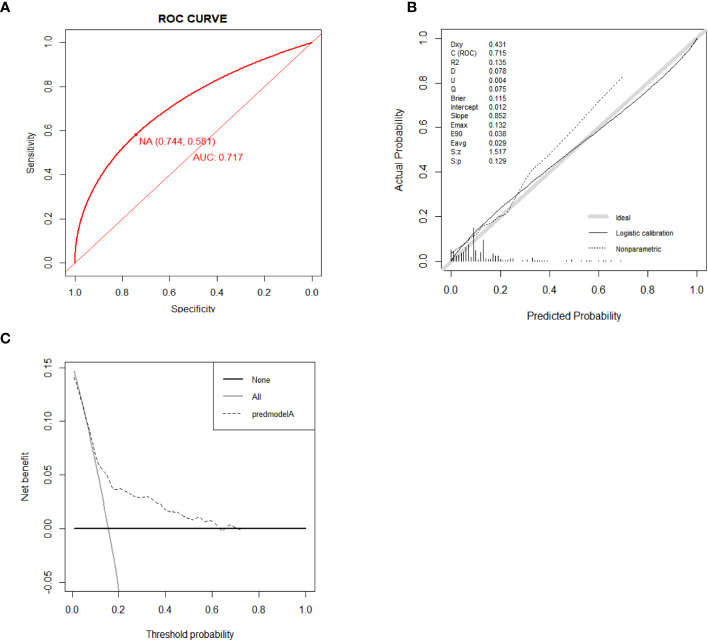
ROC curve for external validation of the all-cause early death nomogram **(A)**. Calibration curve for external validation of the all-cause early death nomogram **(B)**. DCA curves for external validation of the all-cause early death nomogram **(C)**.

The results of external verification were also satisfactory. As shown in the ROC curve, the AUC value of the nomogram was 0.716 (95% CI, 0.633-0.798), indicating a good degree of accuracy in its predictions (**Figure 7A**). The nomogram’s calibration curve demonstrated high consistency (**Figure 7B**). The findings derived from the DCA curves suggest that the new model demonstrates a favorable net benefit and possesses a high level of clinical utility (**Figure 7C**).

## Discussion

It is well recognized that there exists a strong correlation between advanced age and the incidence of cancer (27, 28). Among the 28,111 senior individuals included in the study, a total of 3,500 (12.45%) patients experienced early death. Early death in ECRC patients may be caused by tumor or other conditions including heart disease, respiratory failure, etc. Regrettably, there aren’t many research on early death in ECRC patients, despite the fact that many people have studied CRC or stage IV ECRC patients (15, 22, 29, 30). This is the first time that high-risk variables for early death in ECRC patients have been systematically analyzed, and two nomograms has been developed to forecast the likelihood of early death for this special group.

In our study, the majority of ECRC patients (69.68%) who experienced early death died of cancer. Hence, to enhance the survival rate of this particular group, it is imperative to make precise assessments of the risk of early death in ECRC patients at any time and develop tailored therapeutic strategies for timely intervention in tumor management. The issue is effectively addressed by the implementation of nomograms, which provide a precise estimation of the likelihood of early death among said individuals. Because each patient has a unique physical condition, the cumulative effect from the nomogram must be far better than the predictive effect of individual variables. In contrast to the TNM system, nomogram offer a more comprehensive and systematic approach to assessing prognostic risk factor through combining multiple variables other than TNM staging. Indeed, nomogram have been utilized as a novel predictive model in clinical management within the realm of studying other malignant pathologies (20, 31, 32).

This study observed that seniors in the T4 stage had a greater chance of early death. The reason for this phenomenon as follows: the T stage reflects the depth of tumor infiltration, and at the T4 stage, the tumor may break through the serosa and has the opportunity to cause abdominal dissemination or implantation metastasis (33). In addition, patients in the N2, N1, or M1 stages also have a higher probability of early death.

Prior research has demonstrated that the prognosis of CRC was associated with the primary site of tumor occurrence (34). Left and right colon and rectum tumors differ histologically and molecularly, especially in embryonic origin, metastatic pattern, and therapeutic target composition (35, 36). The location of the original tumor is essential when considering prognosis. Another research found that right-sided colon cancer patients had a different prognosis than left-sided individuals (37). However, according to our study, there appears to be no significant correlation between the initial location of the tumor and early death among patients with ECRC. Due to its retrospective methodology, this research may have selection bias. Further prospective studies are required to corroborate this finding.

The effect of surgery on the prognosis of ECRC patients has been controversial. As comorbidities are more common in the older population than in the younger one, seniors usually have lower surgical tolerance and the risks of surgery exceed the benefits (38–40). On the other hand, surgery is still the mainstay of CRC treatment. Because the proportion of patients who pass away as a direct result of surgical failure is rare, and the permanent disability associated with surgical treatment is only present in elderly patients who are weak (11, 41). Age is not a contraindication to surgery and, similar to younger CRC patients, ECRC patients can benefit from surgery (42, 43). The results of the present study suggest surgery may improve survival and reduce early death risk.

Diagnoses for most ECRC patients (50–60%) occur at the metastatic stage (41). It is necessary to consider whether chemotherapy should be administered to this elderly population compared with youngers.Patients over the age of 75 are mainly associated with comorbidities and have a lower capacity for bone marrow regeneration (44). Previous research has demonstrated, however, that elderly patients with stage III colon cancer can attain the same advantages from chemotherapy without an increase in adverse effects (4). For example, preoperative chemotherapy has demonstrated a promising effect on reducing the stage of locally advanced rectal cancer patients who aged 70 years, and it also increases the possibility of retaining anal sphincter (45). Our findings support the argument that patients with ECRC should continue to get adjuvant or palliative care, and that for those ECRC patients who can tolerance chemotherapy, it is critical to actively assess their physical status in order to choose the best chemotherapy approach.

Radiotherapy has become an important component of colorectal cancer treatment, frequently used to treat patients who are unable to have surgery due to aging or poor overall condition, advanced or recurrent patients, or as a supplement to standard treatment (46). For patients with locally advanced rectal cancer, preoperative radiotherapy is more effective in reducing tumor size, increasing the chance of retaining the anal sphincter, and reducing local recurrence (47). Some radiation therapy-related symptoms, such as long-term intestinal dysfunction and even fecal incontinence, might, nevertheless, considerably affect patient treatment adherence and quality of life (48, 49). Previous research has indicated that radiotherapy is an independent risk factor for early death of metastatic CRC (50). Our research discovered that getting radiotherapy can lessen the risk of early death of patients with ECRC.

Clinicians may be reluctant to offer necessary treatments to older patients due to a paucity of research in this population, although these treatments are likely to have a positive impact on their health (3). Treating elderly people, however, should not be hindered by their age. This study involved a retrospective analysis of 28111 elderly patients from the SEER database. We discovered that patients who did not receive any treatment had an early mortality rate of 47.85%. Patients who underwent surgical treatment alone had a lower early mortality rate of 14.34%. Similarly, patients who received only chemotherapy had an early mortality rate of 21.91%, while those who received only radiotherapy had a rate of 17.24%. Notably, patients who received all three treatments had early mortality rate of 1.25%. Hence, age alone should not be used to judge how to treat the elderly. Thoroughly evaluating the patient’s physical condition and promptly providing personalized programs are essential for improving survival rates and preventing early mortality.

According to our research, liver, bone, and brain metastases are linked to early death in patients with ECRC. Many literatures reported that at the time of their first diagnosis, 20% of ECRC patients had metastases, and the liver was identified as the most prevalent metastatic organ when compared to other organs (51). One study reported that more than 50% of CRC patients developed colorectal cancer liver metastases during the time of their disease, ultimately leading to death in more than two-thirds of patients with only a few could be cured by active treatment (52). It has been demonstrated that intervention at the early stages of liver metastasis, (such as the metastatic and implantation stages), would be more beneficial to improve patients’ survival (53). However, compared with liver metastasis, bone metastasis from CRC is relatively rare, which was reported to occur in approximately 10-15% of CRC patients. When people with CEC developed bone metastases, their median survival was 7-18 months, with a 5-year survival rate of fewer than 5% (54). The reason for the poor prognosis of patients with bone metastasis is that the majority of patients afflicted with bone metastasis frequently have concurrent lung or liver metastasis (55). The median survival after diagnosis of brain metastasis ranged from 2.6 to 7.4 months, and only a very small number of patients survived longer than 1 year (56). As with bone metastasis, because of the blood-brain barrier, when tumor cells invade the brain, there are already metastatic tumors present in other parts of the body (57). Primary tumor cells spreading to distant organ can damage several organ functions. Furthermore, there is a decline in organ functioning in the elderly population when compared to younger people. As a result, patients with ECRC are more likely to die prematurely due to distant spread of tumor cells. It is crucial to actively implement multiple disciplinary treatments (MDT) for elderly patients with metastases from CRC in order to determine the optimum course of treatment, lessen their suffering, and extend their survival.

Observing the nomogram, one will discern that on the horizontal axis at the top, each variable has a matching score, which converts the risk of each factor into a numerical number. By summing the scores of each factor, the total point is computed. Finally, draw a vertical line on the top horizontal axis marked with “point” and cross it with the bottom horizontal axis marked with “probability” to establish the possibility of early death. Moreover, what needs to be declared is not every total point would have a corresponding probability. For example, **Figure 3** show that the minimum value of the bottom horizontal axis marked with “probability” is 0.1, and the corresponding “point” is around 160. When the total score is less than160, the probability of early death is less than 10%, and the probability is not displayed; when the total score is greater than 160, the patient is at risk of early death, and the corresponding probability is obtained.

In our study, these nomograms directly quantify the risk of early death in ECRC patients and can forecast it in real time, including those who had no therapy at diagnosis and those who did. The illustrative case involves a married white female patient, aged 76, who possessed medical insurance, received a diagnosis of rectal cancer. The cancer is classified as Grade III, with a TNM stage of T3N1M0 and tumor size larger than 5cm. Notably, this patient has expressed a refusal to undergo any form of treatment. Currently, it is possible to estimate the likelihood of her mortality resulting from rectal cancer within a three-month timeframe by employing our nomogram, yielding an approximate value of 40%. If the patient undergoes surgical intervention, her likelihood of mortality due to rectal cancer within a three-month period is approximately 10%. Similarly, in the case of an elderly colorectal cancer patient who has not received chemotherapy but has received surgical treatment, tailored treatment recommendations can be made by estimating the likelihood of early death in the event of receiving or not receiving chemotherapy using our nomogram. Also, due to the nomograms’ simple and intuitive nature, doctor-patient conflicts brought on by early death can be efficiently reduced. It is interesting that these nomograms can also be used as a guide for follow-up, making ECRC patients’ long-term care easier.

Although this study has a number of advantages, its possible drawbacks should also be taken into account. First, despite its enormous sample size, the SEER database lacks descriptions of some key information, such as comorbidities, peritoneal metastasis, and how to resolve duplications (for example, same patients with recurrences). Furthermore, neoadjuvant therapy and sole or complementary treatments have not been recorded separately. Second, due to retrospective design of this study, selection bias might affect the results. Third, the predictive model under study is intended to be used at various medical centers around the world, where there are considerable variances in clinical and pathological variables between patients. However, during the process of external validation, we just included the Chinese population, resulting in insufficient case sources and sample size for our external validation cohort. Hence, it is necessary to obtain extensive samples from various cohorts across the globe in order to improve the external validation.

## Conclusion

In conclusion, this study identified risk factors for the early death of ECRC patients, including gender, race, marital status, primary site, tumor size, differentiation grade, histologic type, T stage, N stage, M stage, surgery, chemotherapy, brain metastasis, bone metastasis, lung metastasis, and liver metastasis. Based on these variables, two easy-to-use nomograms were established to forecast the probability of early death, which would contribute significantly to the enhancement of clinical decision-making and the formulation of personalized treatment approaches for this particular population.

## Data availability statement

Publicly available datasets were analyzed in this study. This data can be found here: http://seer.cancer.gov/seerstat.

## Ethics statement

The studies involving humans were approved by China-Japan Union Hospital of Jilin University. The studies were conducted in accordance with the local legislation and institutional requirements. Written informed consent for participation was not required from the participants or the participants’ legal guardians/next of kin in accordance with the national legislation and institutional requirements.

## Author contributions

QW: Writing – original draft, Writing – review & editing. KS: Data curation, Investigation, Writing – review & editing. BF: Data curation, Funding acquisition, Investigation, Writing – review & editing. HL: Data curation, Investigation, Writing – review & editing. RL: Data curation, Investigation, Writing – review & editing. ZW: Validation, Writing – review & editing. MW: Validation, Writing – review & editing. ZX: Methodology, Supervision, Writing – review & editing.
